# Supplementary material for: An Integrative Transcriptomics and Proteomics Approach to Identify Putative Genes Underlying Fruit Ripening in Tomato near Isogenic Lines with Long Shelf Life
Source: Plants (Basel). 2023 Jul 29;12(15):2812. doi: 10.3390/plants12152812 (PMC10421356; doi:10.3390/plants12152812)
Supplement: Supplementary file 1 [file plants-12-02812-s001.zip › SupplementaryMaterial_1.pdf]

SUPPLEMENTARY FIGURES

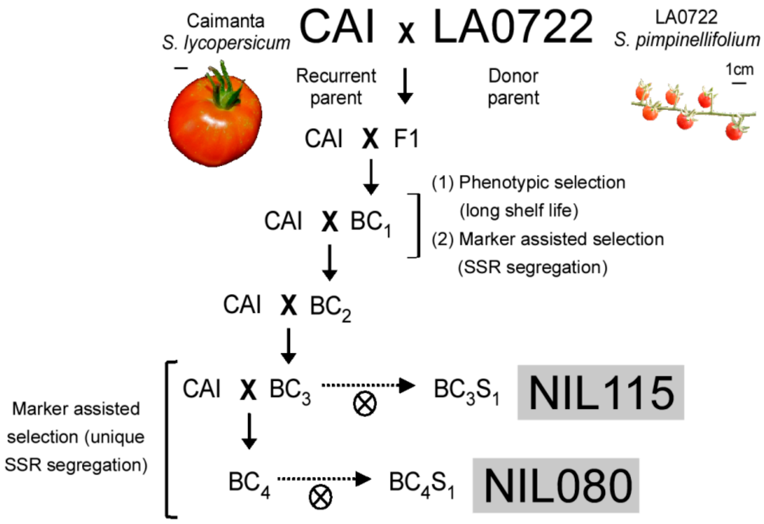

Figure S1 Breeding **s**cheme of plant material used in this study. Grey-squared genotypes were used in this study. NIL115 corresponds to BC_3S_1 individuals carrying a homozygous wild introgression in the SSR115 *locus* (SL4.0ch05:2,779,337) and NIL080 corresponds to BC_4S_1 individuals carrying a homozygous wild introgression in the SSR080 *locus* (SL4.0ch11:2,309,947).

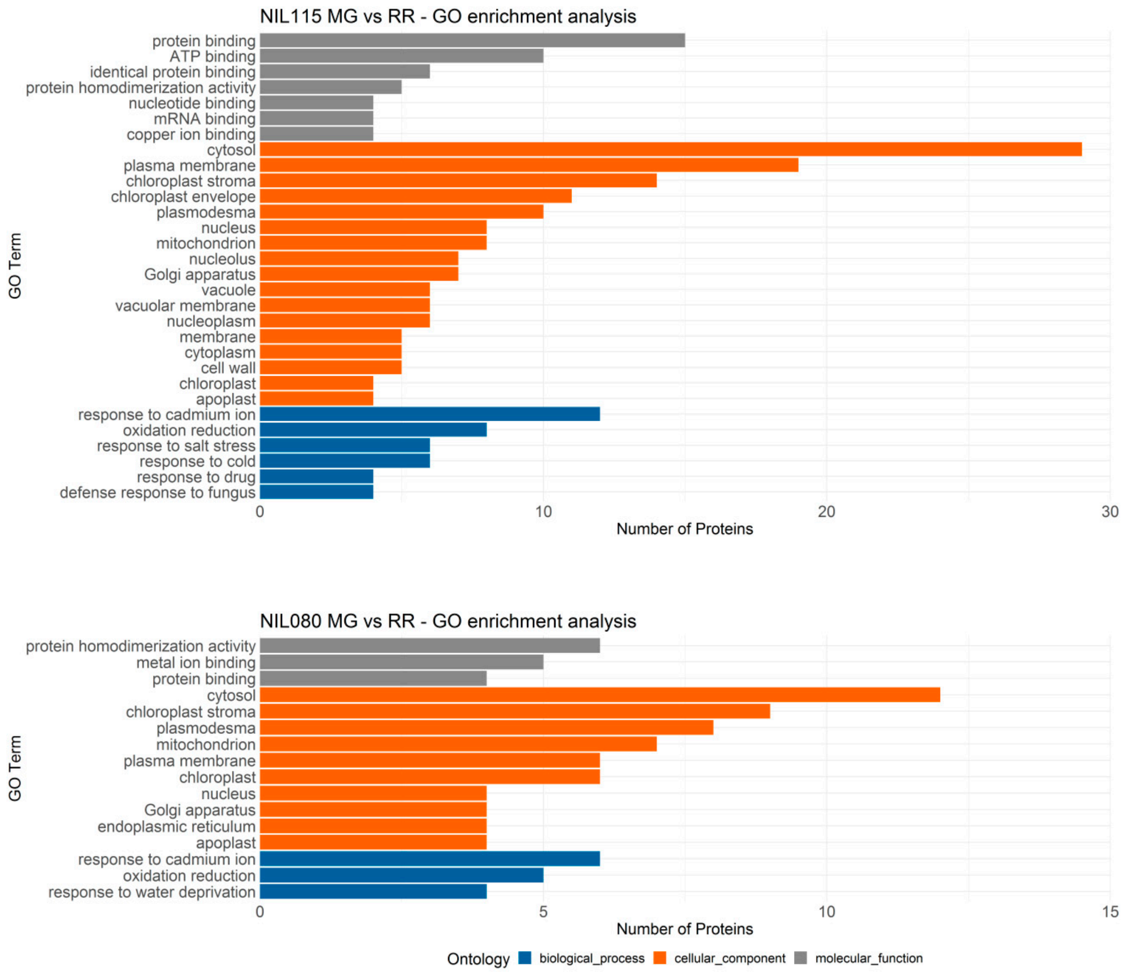

Figure S2 Quantitative protein results from the Plant GO Slim of agriGO v2.0 annotation of red ripe (*RR*) stage compared with mature green (*MG*) stage in the lines NIL115 and NIL080.

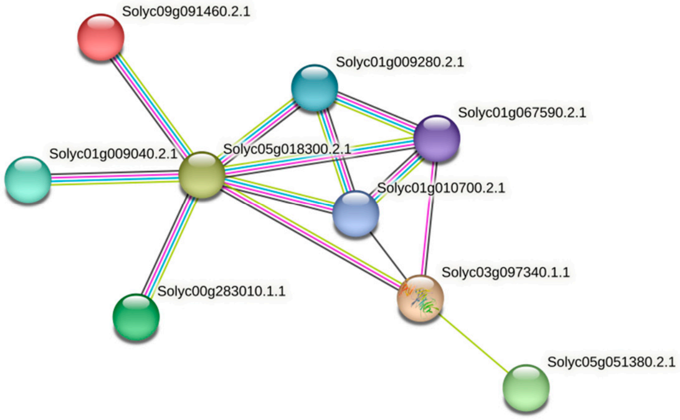

Figure S3 Protein-protein interaction network of *Solyc09g091460* and *Solyc05g051380* in NIL115 generated using the STRING database. The nodes represent individual proteins and the edges (connecting lines) reflect the functional associations derived from various sources. The network includes experimentally determined interactions (pink edges), co-expression (black edges), interaction from curated databases (light-blue edges) and textmining predictions (green edges).
